# Supplementary material for: The Systems Biology Research Tool: evolvable open-source software
Source: BMC Syst Biol. 2008 Jun 29;2:55. doi: 10.1186/1752-0509-2-55 (PMC2446383; doi:10.1186/1752-0509-2-55)
Supplement: Additional file 1 — SBRT Archive. An archive of the current version of the Systems Biology Research Tool. [file 1752-0509-2-55-S1.zip › sbrt-1.4.0/doc/developers_guide/api/sbrt/shell/io/TextOutputFile.html]

TextOutputFile


|  |  |  |
| --- | --- | --- |
| **Overview** \| **Package** \| **Class** \| **Use** \| **Tree** \| **Deprecated** \| **Index** \| **Help** | |  |
| **PREV CLASS**   **NEXT CLASS** | **FRAMES**    **NO FRAMES**     **All Classes** |  |
| SUMMARY: NESTED \| FIELD \| CONSTR \| METHOD | DETAIL: FIELD \| CONSTR \| METHOD |  |


---


## sbrt.shell.io Class TextOutputFile<T>

```
java.lang.Object
  sbrt.shell.io.TextOutputFile<T>
```

**Type Parameters:**: `T` - the type contained in this type of output file.

**All Implemented Interfaces:**: ResultsManager<T>, OutputFile<T>, SbrtFile

---

``` public class TextOutputFile<T> extends java.lang.Object implements OutputFile<T> ```

This class is used to represent output files containing
plain text.

**Author:**
:   This class was written and documented by
    Jeremiah Wright while in the Wagner lab.

---

**Constructor Summary**

`TextOutputFile(java.lang.String fileName,
FileFormat fileFormat,
Formatter<T> lineFormat)`
  
          Constructs a new text output file using the provided
arguments.

`TextOutputFile(java.lang.String fileName,
Formatter<T> lineFormat)`
  
          Constructs a new text output file using the provided
arguments.


| **Method Summary** | |
| --- | --- |
| `<S extends T> void` | `addData(S results)`             Adds data to this output file. |
| `void` | `breakPoint()`             Writes a line of `#` symbols to this file. |
| `void` | `close()`             Closes this file. |
| `protected  void` | `finalize()`             Disposes of system resources associated with this file when called by the garbage collector. |
| `static TextOutputFile<java.lang.String>` | `getBasic(java.lang.String fileName)`             Returns a basic text output file. |
| `static TextOutputFile<java.lang.String>` | `getBasic(java.lang.String fileName, FileFormat fileFormat)`             Returns a basic text output file with the provided file format. |
| `java.lang.String` | `getCanonicalName()`             Returns the canonical name of this file. |
| `Formatter<T>` | `getLineFormat()`             Returns the format used to write lines to this file. |
| `java.lang.String` | `getName()`             Returns the canonical name of this file. |
| `java.lang.String` | `getOriginalName()`             Returns the name of this file. |
| `boolean` | `isClosed()`             Indicates if this file has already been closed. |
| `static boolean` | `isValidFormat(FileFormat fileFormat)`             Indicates if the provided file format is valid for a text output file. |
| `protected  void` | `writeLine(java.lang.String line)`             Writes the provided string to this output file. |

**Methods inherited from class java.lang.Object**

`clone, equals, getClass, hashCode, notify, notifyAll, toString, wait, wait, wait`

**Constructor Detail**

### TextOutputFile

```
public TextOutputFile(java.lang.String fileName,
                      Formatter<T> lineFormat)
```

:   Constructs a new text output file using the provided
    arguments.

    **Parameters:**: `fileName` - the name of the file to be created.: `lineFormat` - the format used for writing formatted data strings to this file.

---


### TextOutputFile

```
public TextOutputFile(java.lang.String fileName,
                      FileFormat fileFormat,
                      Formatter<T> lineFormat)
```

:   Constructs a new text output file using the provided
    arguments.

    **Parameters:**: `fileName` - the name of the file to be created.: `fileFormat` - the file format used to write data to this file.: `lineFormat` - the format used for writing formatted data strings to this file.


**Method Detail**

### isValidFormat

```
public static boolean isValidFormat(FileFormat fileFormat)
```

:   Indicates if the provided file format is valid for
    a text output file.

    :   **Parameters:**: `fileFormat` - the file format to test. **Returns:**: `true` if the provided file format is valid for a text output file; `false` otherwise.

---


### getBasic

```
public static TextOutputFile<java.lang.String> getBasic(java.lang.String fileName)
```

:   Returns a basic text output file.

    :   **Parameters:**: `fileName` - the name with which to name the file. **Returns:**: a basic text output file.

---


### getBasic

```
public static TextOutputFile<java.lang.String> getBasic(java.lang.String fileName,
                                                        FileFormat fileFormat)
```

:   Returns a basic text output file with the provided
    file format.

    :   **Parameters:**: `fileName` - the name of the file to be created.: `fileFormat` - the file format with which to write this file. **Returns:**: a basic text output file with the provided file format.

---


### getName

```
public final java.lang.String getName()
```

:   Returns the canonical name of this file.

    :   **Specified by:**: `getName` in interface `ResultsManager<T>`
    :   **Returns:**: the canonical name of this file.

---


### getOriginalName

```
public final java.lang.String getOriginalName()
```

:   Returns the name of this file.

    :   **Specified by:**: `getOriginalName` in interface `SbrtFile`
    :   **Returns:**: the name of this file.

---


### getCanonicalName

```
public final java.lang.String getCanonicalName()
```

:   Returns the canonical name of this file.

    :   **Specified by:**: `getCanonicalName` in interface `SbrtFile`
    :   **Returns:**: the canonical name of this file. **See Also:**: `File.getCanonicalPath()`

---


### isClosed

```
public final boolean isClosed()
```

:   Indicates if this file has already been closed.

    :   **Specified by:**: `isClosed` in interface `ResultsManager<T>` **Specified by:**: `isClosed` in interface `SbrtFile`
    :   **Returns:**: `true` if this file is closed; `false` othewise. **See Also:**: `ResultsManager.close()`

---


### getLineFormat

```
public Formatter<T> getLineFormat()
```

:   Returns the format used to write lines to this
    file.

    :   **Returns:**: the format used to write lines to this file.

---


### close

```
public void close()
```

:   Closes this file. If this file is already closed, then
    invoking this method has no effect.

    :   **Specified by:**: `close` in interface `ResultsManager<T>` **Specified by:**: `close` in interface `SbrtFile`
    :   **Throws:**: `ApplicationException` - if an I/O error occurred while closing this file.

---


### finalize

```
protected void finalize()
                 throws java.lang.Throwable
```

:   Disposes of system resources associated with this file
    when called by the garbage collector.

    :   **Overrides:**: `finalize` in class `java.lang.Object`
    :   **Throws:**: `java.lang.Throwable` - the Exception raised by this method. **See Also:**: `Object.finalize()`

---


### breakPoint

```
public void breakPoint()
```

:   Writes a line of `#` symbols to this file.

    :   **Specified by:**: `breakPoint` in interface `ResultsManager<T>`
    :   **Throws:**: `java.lang.IllegalStateException` - if `close()` has already been called.

---


### addData

```
public <S extends T> void addData(S results)
```

:   Adds data to this output file. The provided data
    is formatted using this output file's line
    format (see getLineFormat()) and
    followed by a system-dependent new line character.

    :   **Specified by:**: `addData` in interface `ResultsManager<T>`
    :   **Type Parameters:**: `S` - a type extending the primary data type contained in this output file. **Parameters:**: `results` - the results, or data, to be added to this output file. **Throws:**: `ApplicationException` - if an I/O error occurs while writing data to this file.: `java.lang.IllegalStateException` - if `close()` has already been called.: `java.lang.NullPointerException` - if the provided argument is `null`.

---


### writeLine

```
protected final void writeLine(java.lang.String line)
```

:   Writes the provided string to this output file.
    A system-dependent new line character is automatically
    appended to the end of the provided string.

    :   **Parameters:**: `line` - the line to write to this file. **Throws:**: `ApplicationException` - if an I/O error occurs while writing data to this file.: `java.lang.IllegalStateException` - if `close()` has already been called.


---


|  |  |  |
| --- | --- | --- |
| **Overview** \| **Package** \| **Class** \| **Use** \| **Tree** \| **Deprecated** \| **Index** \| **Help** | |  |
| **PREV CLASS**   **NEXT CLASS** | **FRAMES**    **NO FRAMES**     **All Classes** |  |
| SUMMARY: NESTED \| FIELD \| CONSTR \| METHOD | DETAIL: FIELD \| CONSTR \| METHOD |  |


---
